# Fluctuations in local and widespread mechanical sensitivity throughout the migraine cycle: a prospective longitudinal study

**DOI:** 10.1186/s10194-020-1083-z

**Published:** 2020-02-14

**Authors:** Gwendolyne G. M. Scholten-Peeters, Michel W. Coppieters, Tom S. C. Durge, René F. Castien

**Affiliations:** 1 Faculty of Behavioral and Movement Sciences, Vrije Universiteit Amsterdam, Amsterdam Movement Sciences, Van der Boechorststraat 9, 1081 BT Amsterdam, The Netherlands; 2 The Hopkins Centre, Menzies Health Institute Queensland, Griffith University, Brisbane & Gold Coast, Australia; 3 Healthcare Centre Haarlemmermeer, Hoofddorp, The Netherlands; 4 Amsterdam Public Health Research Institute, VU University Medical Center, Amsterdam, The Netherlands

**Keywords:** Headache, Central sensitization, Rehabilitation, Disability, Pain medicine, Pain sciences, Musculoskeletal health, Allodynia

## Abstract

**Background:**

People with migraine have localised (i.e., cephalic) mechanical sensitivity. There is uncertainty regarding widespread (i.e., extra-cephalic) mechanical sensitivity and variations in mechanical sensitivity throughout the migraine cycle. Therefore, this study aimed (1) to comprehensively assess mechanical sensitivity in both cephalic and extra-cephalic regions during the preictal, ictal, postictal and interictal phases; and (2) to compare these findings with mechanical sensitivity at corresponding time-points and locations in healthy participants.

**Methods:**

According to sample size calculations, 19 people with migraine and 19 matched healthy volunteers participated in a prospective longitudinal study. Pressure pain thresholds were evaluated in three cephalic regions (temporalis, upper trapezius and C1 paraspinal muscles) and two extra-cephalic regions (extensor carpi radialis and tibialis anterior muscle) with a digital algometer during the four phases of the migraine cycle in people with migraine and at corresponding intervals and locations in healthy participants. Linear mixed model analyses with a random intercept were used.

**Results:**

People with migraine had increased mechanical sensitivity in cephalic and extra-cephalic regions in all phases of the migraine cycle compared to healthy participants. Furthermore, this mechanical sensitivity was more severe in the preictal, ictal and postictal phase compared to the interictal phase in cephalic and extra-cephalic regions.

**Conclusion:**

People with migraine have localised as well as widespread mechanical sensitivity compared to healthy participants. This sensitivity is even more pronounced immediately before, during and after a migraine attack.

## Introduction

A migraine attack consists of four phases, which vary per patient with respect to duration, content and severity [1]. The preictal phase of an imminent attack usually lasts several hours and can be accompanied by an aura; a neurological phenomenon of unilateral flashes, spots or temporary vision problems [2]. The throbbing headache starts in the ictal phase and typically lasts for 4 to 72 h, followed by the postictal phase in which the intensity of the headache decreases and mood changes, exhaustion and concentration problems may be experienced. The interictal phase is the phase between two consecutive migraine attacks without headache or other symptoms [1].

Although the knowledge of the mechanisms of migraine increased in recent years, the pathophysiology is still far from being fully understood [3–8]. It is assumed that central sensitization plays an important role during a migraine attack [7, 8]. Normally, supraspinal structures, such as the rostroventral medulla, periaqueductal grey, and hypothalamus can inhibit the trigemino-cervical complex [4–6]. A recent study, however, showed that adolescents and first-degree relatives with migraine have similar supraspinal inhibition capacity as healthy participants in the interictal phase despite differences in local and widespread sensitivity [9]. But other studies revealed that people with medication overuse, episodic and chronic migraine show failed supraspinal control of pain [10–12]. Failure of supraspinal control may result in loss of inhibition and hyperexcitability of trigeminovascular neurons [13] leading to dysregulation of antinociceptive processing with mechanical or thermal/or thermal hyperalgesia sensitization [14].

Recent reviews show that people with migraine have significantly higher mechanical sensitivity expressed by lower pressure pain thresholds than healthy participants in the cephalic region during the interictal phase, but there is inconsistent evidence for differences in mechanical sensitivity in the extra-cephalic region [15–17]. Moreover, most studies are performed in the interictal phase, with only a small number of studies also including the ictal phase; and none of the included studies measured mechanical sensitivity in all four phases of the migraine cycle in the cephalic and extra-cephalic region [15, 16]. A recent study, not included in the reviews, assessed cyclic changes in mechanical sensitivity in the cephalic region in people with migraine, and found no statistically significant differences between the interictal, preictal and ictal phase [18].

Based on the hypothesis that people with migraine have established central sensitization which may exacerbate due to extra temporary failure of supraspinal inhibition in addition to possible peripheral mechanisms [19, 20], it is expected that sensitization is a fluctuating phenomenon with increasing mechanical sensitivity from the interictal to the preictal phase, and subsequently to the ictal phase. However, in which phase(s), in which regions [cephalic and/or extra-cephalic) and to what extent mechanical sensitivity fluctuates in people with migraine compared to healthy participants is unknown. Therefore, this study aimed (1) to comprehensively assess mechanical sensitivity during the preictal, ictal, postictal and interictal phases in people with migraine in both cephalic and extra-cephalic regions, and (2) assess differences in mechanical sensitivity between people with migraine and healthy participants in both cephalic and extra-cephalic regions at corresponding time points.

## Methods

### Design

A prospective longitudinal observational study within and between people with migraine and healthy participants. The study was approved by the Medical Ethical Committee of the VU Medical Centre, Amsterdam, The Netherlands (METC-2015-551) and performed according to the Declaration of Helsinki 2013. The study is reported according to the STROBE guidelines [21].

### Participants and setting

People with migraine were recruited from primary healthcare centres between April 2017 and April 2018. During the same period, healthy participants were recruited from the general public via advertisements in local newspapers and social media. Eligibility criteria for people with migraine were: a medical diagnosis of migraine (i.e., migraine diagnosed by a general practitioner according to the International Classification of Headache Disorders (ICDH- III) [22], aged between 18 and 65 years and Dutch or English speaking. Exclusion criteria were: other types of headaches such as medication overuse headache, head or neck complaints within 2 months prior to the measurements, musculoskeletal painful conditions, psychiatric conditions, malignancy or other neuropathic pain states. Furthermore, participants who received treatment for headache 48 h before the measurements or those who received botulinum toxin injections were also excluded. Participants were not allowed to use analgesic medication within 24 h before the measurements. Healthy participants were people without current pain or a history of chronic pain, neurological disorders or headache in the last 12 months. Healthy participants were matched for age and sex. All participants provided written informed consent prior to participating in the study.

### Baseline measurement (interictal phase)

Baseline measurements were performed in the interictal phase and consisted of completion of various questionnaires and the assessment of mechanical sensitivity via determination of pressure pain thresholds (PPTs). All measurements were conducted at a primary healthcare centre. A questionnaire was completed to obtain sociodemographic information, medication use and migraine characteristics. The six-item Headache Impact Test (HIT-6) [23] was used to measure the impact of headache on daily functioning. Scores on the HIT-6 range from 36 to 78. The HIT-6 is a reliable (ICC = 0.77) and valid tool to discriminate headache impact in a migraine population [23]. The Central Sensitization Inventory was used to gain insight into the degree of central sensitization [24]. The inventory has good test-retest reliability in people with chronic pain (ICC = 0.88) and healthy participants (ICC = 0.91) [24]. It consists of 25 items, and the score ranges from 0 to 100. The Allodynia Symptom Checklist (ASC-12) is a 12-item validated questionnaire to identify cutaneous allodynia with a score ranging from 0 to 12 [25]. Additionally, all participants rated their headache pain intensity before each measurement on a numeric pain rating scale (NPRS). The scale ranges from 0 to 10, and is valid and reliable in several patient populations [26].

### Mechanical sensitivity

PPTs were measured bilaterally at five test locations in a fixed, cyclic order with a 20 s interval between the measurements: 1) temporalis muscle (i.e., 1 cm lateral to the external angle of the orbit) [27], 2) paraspinal muscles C1 (i.e., 2 cm lateral of the midline of the neck, below the occipital bone), 3) upper trapezius muscle (i.e., midpoint between acromion and spinous process C7) [16, 28], 4) extensor carpi radialis muscle (i.e, at 1/3 of the length of the underarm, distal to the elbow) and 5) tibialis anterior muscle (i.e., at 1/3 of the length of the lower leg, distal to the knee). The first three test locations are cephalic regions and the latter two are extra-cephalic regions. In totally, PPTs were determined three times at the same location; first on the dominant side of the migraine (defined as the most painful side of the current attack) and subsequently on the non-dominant side. In case of bilateral migraine, the most prevailing side of the headache was measured first. The patients’ dominant side was compared with the side of the dominant hand in healthy participants (and the patients’ non-dominant side with the side of the non-dominant hand in healthy participants). Participants were instructed to press a switch when they first felt the sensation of pressure change into pain. PPTs were measured with a digital algometer (Algometer type II, rubber probe area 1cm^2^, Somedic Electronics, Solna, Sweden) with an application rate of 50 kPa per second. The algometer is a valid and reliable (ICC: 0.75–0.95) instrument to measure PPTs [29, 30].

### Follow-up measurements and blinding

PPTs were assessed during the four phases of the migraine cycle. The timing of the sessions was individualized. Patients contacted the research centre when their headache started and were measured within several hours for the preictal phase measurement. The ictal measurement in which they experienced the throbbing headache was routinely scheduled 1 day later and the postictal assessment was individually planned one till 3 days after the ictal measurement depending on individual experiences of ictal phase duration. Healthy participants were measured accordingly with equal time intervals between the measurements as the matched person with migraine.

### Sample size calculation

A sample size calculation was performed based on the effect size and standard deviation (SD) of a study that compared PPTs at the trapezius muscle between people with migraine and healthy participants [31]. Based on a mean difference between groups of 56 kPa, a SD of 68, two-tailed α of 0.05, β of 0.80, 3 follow-up measurements, intra-person correlation coefficient of 0.6 and an anticipated dropout rate of 10%, 19 participants per group were required.

### Statistical analysis

The mean from the three PPT measures at the same location was calculated for the dominant and non-dominant sides. A PPT value more than three standard deviations from the mean of the three measures, was considered as an outlier and was removed. In those cases, the mean was calculated based on the remaining two PPT measures. Participants who did not attend the four measurement sessions were excluded from the statistical analysis. Normality of continuous variables was visually inspected by Q-Q plots, box plots and histograms and checked by the Kolmogorov-Smirnov test. Characteristics were compared both visually and statistically with an independent sample *t* tests or Mann-Whitney *U* tests for continuous variables and Chi square tests for dichotomous variables. Linear mixed model analyses with fixed factor (time), covariate (group) and interaction (time*group) were used to detect differences between the groups at the four time points in the cephalic and extra-cephalic region. A linear mixed model analysis with fixed factor (time) was performed to detect differences within the migraine group between the four phases. A random intercept was chosen to account for the correlated nature of multiple measurements from the same individual. The regression coefficient (B), *p*-value, and confidence intervals (95%CI) were computed for the crude models, as well as for the models that were adjusted for age and baseline PPT values (i.e., PPT values in the interictal phase). A p-value of < 0.05 was considered to be statistically significant. Individual line graphs were created per participant to visualize individual fluctuations throughout the migraine cycle and visualize individual differences in PPTs. SPSS version 25.0 (IBM Corp., Armonk, New York, USA) was used for statistical analysis.

## Results

### Participants

**Fig. 1:**
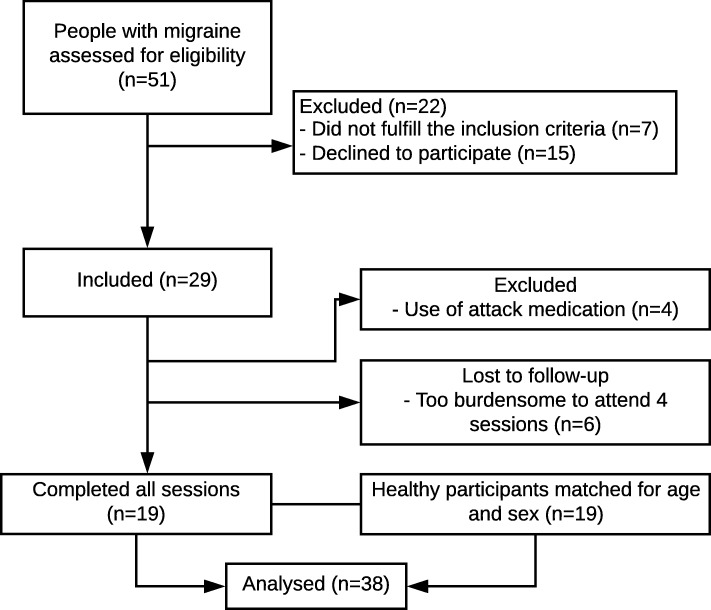
Flow chart of the study

Fifty-one people with migraine were assessed for eligibility criteria of whom 29 commenced the study. Four patients had to be excluded during the study as they used analgesic medication and six patients did not attend one or more follow-up sessions. Nineteen people completed all four measurement sessions. There were no significant differences in participant characteristics between people with migraine who completed all sessions and those who were excluded during the study (*p* > 0.136). Figure 1 provides the flow diagram of the study, and Table 1 summarizes the participant characteristics at baseline.

**Table 1:** Baseline characteristics for people with migraine and healthy participants

	People with migraine (*n* = 19)	Healthy participants (*n* = 19)
Age in years	47.3 (11.7)	47.5 (11.8)
Sex: female (%)	16 (84.2%)	16 (84.2%)
Hand dominance: right (%)	16 (84.2%)	18 (94.7%)
Years with migraine^a^	30.0 (12.0–34.0)	N/A
Migraine days/month^a^	5.0 (4.0–10.0)	N/A
Episodic migraine (%)	14 (73.7%)	
Chronic migraine (%)	5 (23.3%)	
Side of migraine (%)		
*Dominantly left*	7 (36.8%)	N/A
*Dominantly right*	9 (47.4%)	
*Bilateral*	3 (15.8%)	
Aura: yes (%)	6 (31.6)	N/A
Pain intensity (NPRS), interictal phase^a^	0.0 (0.0–1.0)	0.0 (0.0–0.0)
Central Sensitization Inventory	32.7 (11.1)	20.0 (11.2)*
Allodynia Symptom Checklist-12 items	3.4 (2.4)	0.6 (2.1)*
Headache Impact Test-6 item	62.3 (8.9)	44.4 (10.2)*
Pain medication (NSAIDs or paracetamol) used in last month: yes (%)	12 (63.2%)	0
Prophylactic medication used in the last month: yes (%)	8 (42.1%)	0
*Beta-blockers*	7 (36.8%)	
*Antidepressants*	1 (5.3%)	
Triptans used in last month: yes (%)	16 (84.2%)	0

Values are presented as mean (SD) for continuous data and as percentages for categorical data unless stated otherwise. ^a^Data are presented as median and interquartile range. *N/A* not applicable, *NPRS* numeric pain rating scale, *NSAIDs* non steroidal anti-inflammatory drugs. * = Significant difference between groups (*p* <  0.05)

### Changes in pressure pain thresholds throughout the migraine cycle in people with migraine

PPTs in people with migraine were significantly lower in the preictal, ictal and postictal phase compared to the interictal phase in both the cephalic and extra-cephalic regions (Table 2). The strongest decrease in PPTs was found in the ictal phase compared to the interictal phase in the cephalic and extra-cephalic regions. The results at the non-dominant side were comparable with the dominant side in the cephalic regions with significantly lower PPTs in the preictal, ictal and postictal phase compared to the interictal phase (Appendix 1). For the extra-cephalic regions (non-dominant side), significantly lower PPTs were found in the ictal phase compared to the interictal phase, postictal phase (extensor carpi radialis muscle) and preictal phase (tibialis anterior muscle), though not in the preictal phase on the extensor carpi radialis muscle and the postictal phase on the tibialis anterior muscle (Appendix 1).

**Table 2:** Differences in pressure pain thresholds for the preictal, ictal and postictal phase compared to the interictal phase in people with migraine (dominant side)

		Crude			Adjusted^a^		
		Mean	95% CI	*p*-value	Mean	95% CI	*p*-value
Temporalis muscle	Interictal	181.5	159.0–201.0	N/A	163.9	70.4–257.5	N/A
	Preictal	−30.7	−39.9 - -21.5	< 0.001	− 30.7	− 39.9 - -21.5	< 0.001
	Ictal	−60.5	−69.7- -51.3	< 0.001	−60.5	−69.7- -51.3	< 0.001
	Postictal	−16.1	− 25.3- -6.9	0.001	− 16.1	− 25.3- -6.9	0.001
Paraspinal muscles C1	Interictal	225.7	197.1–254.3	N/A	180.0	62.6–297.4	N/A
	Preictal	−28.9	− 40.2- -17.5	< 0.001	− 28.9	−40.2- -17.5	< 0.001
	Ictal	−61.4	−72.8- -50.1	< 0.001	− 61.4	− 72.8- -50.1	< 0.001
	Postictal	−16.1	− 27.5- -4.8	0.006	−16.1	−27.5- -4.8	0.006
Upper trapezius muscle	Interictal	312.2	276.6–347.8	N/A	324.3	175.0–473.6	N/A
	Preictal	−48.0	−60.5- -35.5	< 0.001	−48.0	−60.5- -35.5	< 0.001
	Ictal	−80.4	−92.9- -67.9	< 0.001	−80.4	−92.9- -67.9	< 0.001
	Postictal	−24.0	−36.5- -11.5	< 0.001	−24.0	36.5- -11.5	< 0.001
Extensor carpi radialis muscle	Interictal	237.5	211.6–263.3	N/A	249.1	140.2–358.1	N/A
	Preictal	−12.5	−20.5- -4.4	0.003	−12.5	−20.5- -4.4	0.003
	Ictal	−20.4	−28.5- -12.3	< 0.001	− 20.4	− 28.5- -12.3	< 0.001
	Postictal	−12.9	−20.9- -4.8	0.002	−12.9	−20.9- -4.8	0.002
Tibialis anterior muscle	Interictal	441.4	381.0–501.8	N/A	495.5	243.0–747.9	N/A
	Preictal	−30.1	−50.5- -9.6	0.005	− 30.1	−50.5- -9.6	0.005
	Ictal	−47.9	−68.3- -27.4	< 0.001	−47.9	−68.3- -27.4	< 0.001
	Postictal	−22.5	−42.9- -2.0	0.032	−22.5	−42.9- -2.0	0.032

*CI* confidence interval. Mean PPT (in kPa) compared to the interictal phase; negative values mean lower PPT scores compared to the interictal phase. ^a^Adjusted for age

### Pressure pain thresholds for people with and without migraine

People with migraine showed significantly lower PPTs than healthy participants throughout the four phases of the migraine cycle at all five test locations at both the dominant side (Table 3) and non-dominant side (Appendix 2). There were no outliers.

**Table 3:** Differences in pressure pain thresholds between people with migraine and healthy participants (dominant side)

		Crude			Adjusted^a^		
		Difference	95% CI	*P*-value	Difference	95% CI	*P*-value
Temporalis muscle	Interictal	−91.4	− 125.8 – −57.0	< 0.001	−3.0	−12.5 – 6.5	0.534
	Preictal	− 115.8	− 151.0 – −80.4	< 0.001	−27.4	−36.9 – − 17.9	< 0.001
	Ictal	− 152.9	− 188.1 – − 117.8	< 0.001	− 64.5	−74.0 – − 55.1	< 0.001
	Postictal	−108.0	− 143.2 – −72.8	< 0.001	−19.6	− 29.0 – − 10.1	< 0.001
Paraspinal muscles C1	Interictal	−56.4	− 90.7 – − 22.2	0.001	− 3.2	− 13.0 – 6.7	0.525
	Preictal	− 86.4	− 121.4 – − 51.3	< 0.001	−33.1	−42.9 – − 23.3	< 0.001
	Ictal	−120.3	−155.3 – − 85.3	< 0.001	−67.1	−76.9 – − 57.2	< 0.001
	Postictal	− 75.1	− 110.1 – −40.1	< 0.001	−21.8	−31.7 – − 12.0	< 0.001
Upper trapezius muscle	Interictal	−46.9	− 84.9 – − 9.0	0.016	− 6.1	−17.1 – 4.9	0.277
	Preictal	−92.5	−31.3 – −53.7	< 0.001	−51.6	− 62.6 – −40.6	< 0.001
	Ictal	− 125.7	− 164.5 – − 86.9	< 0.001	−84.9	−95.9 – −73.9	< 0.001
	Postictal	−70.8	−109.6 – − 32.0	0.001	−29.9	−40.9 – −18.9	< 0.001
Extensor carpi radialis muscle	Interictal	−39.3	−72.5 – − 6.1	0.021	−2.6	−11.3 – 6.0	0.548
	Preictal	−51.3	−85.3 – −17.3	0.004	−14.6	−23.3 – −5.9	0.001
	Ictal	−60.2	−94.1 – −26.2	0.001	−23.5	−32.2 – − 14.8	< 0.001
	Postictal	−53.5	− 87.4 – −19.5	0.003	−16.8	−25.5 – −8.1	< 0.001
Tibialis anterior muscle	Interictal	− 111.5	− 181.1 – −41.8	0.002	−1.1	−20.6 – 18.5	0.915
	Preictal	−143.5	− 214.7 – −72.2	< 0.001	−33.1	−52.6 – −13.5	0.001
	Ictal	−162.2	− 233.5 – −91.0	< 0.001	−51.8	−71.4 – −32.3	< 0.001
	Postictal	− 132.5	− 203.7 – −61.3	0.001	−22.1	−41.6 – − 2.5	0.027

Data (in kPa) are presented as mean differences and 95% confidence intervals (95% CI). ^a^Adjusted for age and baseline (i.e., interictal) PPT value

These findings were observed in both the crude and the adjusted analyses, except for the extensor carpi radialis muscle in the preictal phase (non-dominant side).

### Individual pressure pain thresholds scores over time

**Fig. 2:**
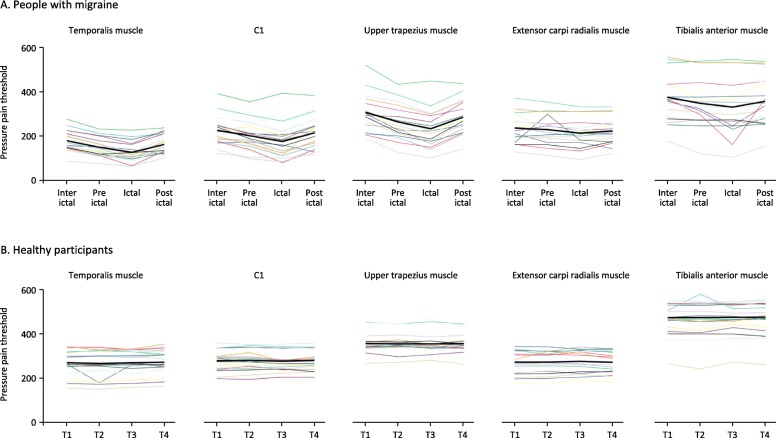
Individual pressure pain thresholds for the three cephalic regions (temporalis muscle, C1 and upper trapezius muscle) and two extra-cephalic regions (extensor carpi radialis and tibialis anterior muscle) on the dominant side for the four phases of the migraine cycle for people with migraine. Healthy participants were measured at corresponding time intervals (T1 ≙ interictal, T2 ≙ preictal, T3 ≙ ictal, T4 ≙ postictal). Thin lines represent individual participants. The bold black line represents the mean

To visualize individual fluctuations in PPT patterns throughout the migraine cycle, line graphs per participant were created in the cephalic and extra-cephalic regions in people with migraine and matched healthy participants at the dominant side (Fig. 2). Similar patterns with decreasing PPTs from the interictal to the preictal and subsequent to the ictal phase were observed in people with migraine and were most pronounced in the cephalic region at the dominant side. But large individual differences in magnitude and fluctuations in mechanical sensitivity were observed. No cyclic changes were visually and statistically detected in the healthy participants (*p* > 0.289).

## Discussion

Throughout the four phases of the migraine cycle, people with migraine have mechanical sensitivity in cephalic and extra-cephalic regions compared to healthy participants. This mechanical sensitivity is even more pronounced immediately before (preictal), during (ictal) and after (postictal) a migraine attack.

Our findings illustrate that people with migraine have localised as well as widespread mechanical sensitivity. The presence of widespread bilateral mechanical sensitivity supports the view that central sensitization mechanisms are involved in migraine, in addition to possible peripheral mechanisms as comprehensive mechanism. Mechanical sensitivity in the cephalic regions observed in migraine seem to be related to sensitisation in first-order and second-order neurons in the trigemino-cervical complex. Moreover, sensitisation in third-order neurons in the thalamus and brainstem and fourth-order neurons in the cortex might be responsible for widespread sensitivity and cyclic changes that we found.

The findings of lower PPT values in people with migraine compared to healthy participants in the interictal phase in the cephalic region are in line with previous systematic reviews [15, 16]. A few studies evaluated PPTs in the extra-cephalic region in the interictal phase [32, 33]. One of these studies also found lower PPTs in the interictal phase in extra-cephalic regions (tibialis anterior muscle and second metacarpal) in people with migraine compared to healthy participants [33]. The other study measured PPTs at the forearm in the interictal phase in women with menstrual migraine and found no significant differences compared to healthy participants [32]. A possible mechanism for the extra-cephalic mechanical sensitivity that we found might be hyperexcitability developing along trigeminal pathways or failure of pain-inhibiting thalamo-cortical pathways outside the migraine attack.

The cyclic fluctuations in mechanical sensitivity observed in this study are in contrast with a recent study of 21 people with migraine and 33 healthy participants [18]. The latter study found no significant differences in PPTs in the head region between the interictal, preictal and ictal phases [18]. PPTs were measured in the frontal, temporal and occipital area of the head. The differences in results compared to our study may be explained by the fact that their measurements may not have been performed in the intended phase of the migraine cycle due to pre-planned scheduling of the measurements, differences in statistical analyses (repeated-measures analysis of variance versus linear mixed models accounting for clustered data) and insufficient matching of participants [18].

In people with strictly unilateral migraine, previous research found significant side to side differences in cephalic PPTs in the interictal phase [34]. Other researchers found bilaterally sensitized cephalic PPTs regardless of migraine laterality in women with chronic and episodic headache compared to healthy participants [35].

Changes in cortical motor evoked potentials have been observed in people with episodic migraine [36]. These changes in cortical excitability also fluctuate throughout the migraine cycle and may contribute to our findings. To interpret the altered sensitivity to mechanical pressure in a person with migraine, it is important to realize not only in which phase of the migraine cycle the measurement was performed, but also in which region (cephalic or extra-cephalic) and at which side (dominant or non-dominant) the measurement was conducted.

Measuring PPTs during each of the four phases of a migraine attack was challenging, for both patients with migraine and the assessor. As migraine attacks mostly occur irregularly and occasionally at night, measurement sessions could not be scheduled in advance. All people with migraine were contacted 1 day after the ictal phase measurement session and a tentative meeting was scheduled based on individual experiences of the ictal phase duration. In case of deviations, the patient contacted the research centre and a new measurement session was planned for the next day. Consequently, the time intervals between the measurements were individually adjusted. Corresponding time intervals were used for the matched healthy participants.

Attending four sessions was too burdensome for some patients, especially as they were not allowed to take analgesic medication 24 h before the measurements. Nevertheless, we were able to include the required sample of 19 people with migraine. As our complete cases were comparable with the patients excluded during the study, selection bias seems unlikely.

In line with the protocol, the assessor was blinded to the type of participant and the phase of the migraine cycle. However, clinical signs and symptoms (especially in the ictal phase) often compromised blinding. Because PPT measurements were performed in a standardised manner (including application rate and communication), and because participants rather than the assessor determined the threshold via pressing a hand-held switch, we believe it is unlikely that this compromised our findings.

We did not distinguish between episodic and chronic migraine, and did not create subgroups for people with or without aura as both groups demonstrate lower PPTs compared to healthy participants [15, 17, 35]. Moreover, creating subgroups for migraine was not possible due to the low numbers of people with migraine with aura and those with chronic migraine. Inclusion of different types of migraine may have increased the heterogeneity of the study population and may have decreased the internal validity, but may have increased the generalisability of our findings. For the female participants, no information was recorded about menstrual cycle. However, mechanical sensitivity can also fluctuate throughout the menstrual cycle with higher pain thresholds in the luteal phase compared to the follicular and ovulatory phase in people with migraine and healthy participants without significant differences between the groups [32]. The potential impact of menstrual cycle on our findings cannot be excluded. The use of orally administered prophylactic anti-migraine drugs by eight participants could also have influenced our results. Preclinical research revealed that repeated use of prophylactic treatment, such as beta-blockers, decreased mechanical sensitivity in cephalic and extra-cephalic regions [37, 38]. In other words, this medication use might have dampened rather than increased our observations of increased sensitivity in people with migraine.

We only assessed mechanical sensitivity. Other studies revealed increased sensitivity for thermal pain thresholds throughout the migraine cycle [14, 15]. However, the same fluctuations throughout the migraine cycle have not yet been demonstrated comprehensively for thermal pain thresholds [14] or conditioned pain modulation to assess descending pain inhibition systems.

## Conclusions

People with migraine experience mechanical sensitivity throughout the four phases of the migraine cycle compared to healthy participants. Sensitivity for mechanical pain thresholds fluctuate throughout the migraine cycle in cephalic and extra-cephalic regions at the dominant and non-dominant side of the migraine. Localised as well as widespread mechanical sensitivity is more pronounced immediately before (preictal), during (ictal) and after (postictal) a migraine attack. Our findings support the view that central sensitization mechanisms are involved in people with migraine.

## Data Availability

The datasets used and/or analyzed during the current study are available from the corresponding author on reasonable request.

## Appendix 1

**Table 4:** Differences in pressure pain thresholds for the preictal, ictal and postictal phase compared to the interictal phase in people with migraine (non-dominant site)

		Crude			Adjusted^a^		
		Mean	95% CI	*p*-value	Mean	95% CI	*p*-value
Temporalis muscle	Interictal	178.2	158.1–198.3	N/A	174.0	90.2–257.8	N/A
	Preictal	−29.4	−37.7 – − 21.0	< 0.001	−29.4	− 37.7 – − 21.0	< 0.001
	Ictal	− 52.5	− 60.8 – − 44.1	< 0.001	− 52.5	− 60.8 – − 44.1	< 0.001
	Postictal	−16.7	−25.1 – −8.4	< 0.001	− 16.7	− 25.1 – − 8.4	< 0.001
Paraspinal muscles C1	Interictal	225.5	193.9–257.2	N/A	180.3	49.1–257.8	N/A
	Preictal	−24.8	−35.7 – − 13.8	< 0.001	− 24.8	− 35.7 – − 13.8	< 0.001
	Ictal	−50.9	− 61.8 – − 30.9	< 0.001	− 50.9	− 61.8 – − 30.9	< 0.001
	Postictal	− 10.9	− 21.9 – − 0.004	0.050	−10.9	− 21.9 – - 0.004	0.050
Upper trapezius muscle	Interictal	311.2	274.5–348.0	N/A	310.8	155.8–465.7	N/A
	Preictal	− 44.3	− 55.7 – − 32.9	< 0.001	− 44.3	−55.7 – − 32.9	< 0.001
	Ictal	−75.1	−86.5 – − 63.8	< 0.001	− 75.1	− 86.5 – − 63.8	< 0.001
	Postictal	−22.7	−34.1 – − 11.4	< 0.001	− 22.7	− 34.1 – − 11.4	< 0.001
Extensor carpi radialis muscle	Interictal	238.9	211.0–266.9	N/A	242.5	126.6–358.3	N/A
	Preictal	−6.8	−19.4 – 5.7	0.279	−6.8	−19.4 – 5.7	0.279
	Ictal	−22.4	− 35.0 – − 9.9	0.001	− 22.4	− 35.0 – − 9.9	0.001
	Postictal	−12.7	−25.2 – − 0.2	0.047	− 12.7	− 25.2 – − 0.2	0.047
Tibialis anterior muscle	Interictal	443.3	383.1–503.5	N/A	508.4	257.9–758.9	N/A
	Preictal	−31.7	−53.5 – − 9.9	0.005	− 31.7	− 53.5 – − 9.9	0.005
	Ictal	− 51.9	− 73.7 – − 30.0	< 0.001	− 51.9	− 73.7 – − 30.0	< 0.001
	Postictal	− 21.6	− 43.5 – 0.2	0.052	−21.6	−43.5 – 0.2	0.052

*CI* confidence interval. Mean PPT (in kPa) compared to the interictal phase; negative values mean lower PPT scores compared to the interictal phase. ^a^Adjusted for age

## Appendix 2

**Table 5:** Differences in pressure pain thresholds between people with migraine and healthy participants (non-dominant side)

		Crude			Adjusted^a^		
		Difference	95% CI	*P*-value	Difference	95% CI	*P*-value
Temporalis muscle	Interictal	−93.3	--125.0 – − 61.7	< 0.001	*−*5.3	−14.9 – 4.2	0.271
	Preictal	−118.9	− 150.5 – − 87.2	< 0.001	− 30.9	−40.4 – − 21.4	< 0.001
	Ictal	− 145.3	− 177.0 – − 113.7	< 0.001	− 57.4	−66.9 – − 47.8	< 0.001
	Postictal	−112.2	− 143.8 – − 80.6	< 0.001	− 24.2	−33.8 – − 14.7	< 0.001
Paraspinal muscles C1	Interictal	−53.8	− 90.1– − 17.6	0.004	−0.8	−9.7 – 8.2	0.867
	Preictal	−80.5	− 116.8 – − 44.3	< 0.001	− 27.5	− 36.4 – − 18.5	< 0.001
	Ictal	−102.9	− 139.1– − 66.6	< 0.00	−49.8	−58.7 – − 40.9	< 0.001
	Postictal	−66.3	− 102.5 – − 30.0	< 0.001	−13.2	− 22.1 – −4.3	0.004
Upper trapezius muscle	Interictal	−46.2	−85.5 – − 6.9	0.022	−3.5	−14.8 – 7.8	0.540
	Preictal	−89.4	− 128.7 – − 50.1	< 0.001	−46.7	−58.0 – − 35.4	< 0.001
	Ictal	− 119.7	− 159.0 – − 80.4	< 0.001	−77.0	− 88.3 – − 65.7	< 0.001
	Postictal	−68.5	−107.8 – − 29.2	0.001	−25.8	−37.2 – − 14.5	< 0.001
Extensor carpi radialis muscle	Interictal	−35.1	−69.9 – − 0.4	0.048	−3.0	− 14.7 – 8.8	0.620
	Preictal	−42.0	− 76.8 – − 7.3	0.018	−9.9	−26.6 – 1.9	0.100
	Ictal	−61.1	−95.8 – − 26.3	0.001	−28.9	−40.6 – − 17.1	< 0.001
	Postictal	−47.3	−82.0 – − 12.5	0.008	− 15.1	−26.9 – −3.3	0.012
Tibialis anterior muscle	Interictal	−110.4	− 179.5 – − 41.2	0.002	1.1	−19.5 – 21.7	0.916
	Preictal	−143.0	−212.1 – − 73.8	< 0.001	−31.5	−52.1 – −10.9	0.003
	Ictal	−163.6	− 232.8 – − 94.5	< 0.001	− 52.2	−72.9 – − 31.5	< 0.001
	Postictal	−134.5	−203.7 – − 65.4	< 0.001	−23.1	− 43.7 – −2.5	0.029

CI *confidence interval*. Mean difference in PPTs (in kPa) between people with migraine and healthy participants in the different phases of migraine. ^a^Adjusted for age and baseline PPT value

## Abbreviations

ASC-12: Allodynia symptom checklist 12 items
CI: Confidence interval
HIT-6: Headache impact test 6 items
ICDH: International Classification of Headache Disorders
NPRS: Numeric pain rating scale
PPT: Pressure pain thresholds
SD: Standard deviation
